# Adipose-derived mesenchymal stem cells cultured in serum-free medium attenuate acute contrast-induced nephropathy by exerting anti-apoptotic effects

**DOI:** 10.1186/s13287-023-03553-8

**Published:** 2023-11-22

**Authors:** Mitsuki Kadono, Ayumu Nakashima, Naoki Ishiuchi, Kensuke Sasaki, Yoshie Miura, Satoshi Maeda, Asuka Fujita, Ayano Sasaki, Shogo Nagamatsu, Takao Masaki

**Affiliations:** 1 Department of Nephrology, Hiroshima University Hospital, 1-2-3 Kasumi, Minami-ku, Hiroshima, 734-8551 Japan; 2 Department of Stem Cell Biology and Medicine, Graduate School of Biomedical & Health Sciences, Hiroshima University, 1-2-3 Kasumi, Minami-ku, Hiroshima, 734-8553 Japan; 3 TWOCELLS Company, Limited, 16-35 Hijiyama-Honmachi, Minami-ku, Hiroshima, 732-0816 Japan; 4 Department of Plastic and Reconstructive Surgery, Hiroshima University Hospital, 1-2-3 Kasumi, Minami-ku, Hiroshima, 734-8551 Japan

**Keywords:** Mesenchymal stem cell, Serum-free medium, Acute kidney injury, Contrast-induced nephropathy, Apoptosis, Epidermal growth factor

## Abstract

**Background:**

Contrast-induced nephropathy (CIN) is a major clinical problem associated with acute kidney injury during hospitalization. However, effective treatments for CIN are currently lacking. Mesenchymal stem cells (MSCs) have protective effects against kidney injury by suppressing inflammation and fibrosis. We previously showed that MSCs cultured in serum-free medium (SF-MSCs) enhance their anti-inflammatory and anti-fibrotic effects. However, whether SF-MSCs potentiate their anti-apoptotic effects is unknown. Here, we investigated the effects of SF-MSCs on a CIN mouse model.

**Methods:**

To create CIN model mice, we removed right kidney at first. One week later, the left renal artery was clamped for 30 min to cause ischemia–reperfusion injury, and mice were injected with iohexol. Then the kidney received 10 Gy of irradiation, and MSCs or SF-MSCs were injected immediately. At 24 h post-injection, mice were sacrificed, and their blood and kidneys were collected to evaluate renal function, DNA damage, and apoptosis. In addition, apoptosis was induced in HEK-293 cells by irradiation and cells were treated with conditioned medium from MSCs or SF-MSCs.

**Results:**

Treatment of CIN model mice with SF-MSCs markedly improved renal function compared with MSCs treatment. Cleaved caspase-3 levels and TUNEL-positive cell numbers were strongly suppressed in CIN model mice treated with SF-MSCs compared with the findings in those treated with MSCs. γH2AX levels, a chromosome damage marker, were reduced by MSCs and further reduced by SF-MSCs. In addition, cleaved caspase-3 in irradiated HEK-293 cells was more strongly suppressed by conditioned medium from SF-MSCs than by that from MSCs. Secretion of epidermal growth factor (EGF) was enhanced by culturing MSCs in serum-free medium. Knockdown of EGF by siRNA attenuated the inhibitory effects of SF-MSCs on CIN-induced renal dysfunction and tubular apoptosis.

**Conclusions:**

These findings strongly suggest that SF-MSCs improve CIN in model mice by exerting anti-apoptotic effects in a paracrine manner. Thus, SF-MSCs represent a potential novel therapy for CIN.

**Graphical Abstract:**

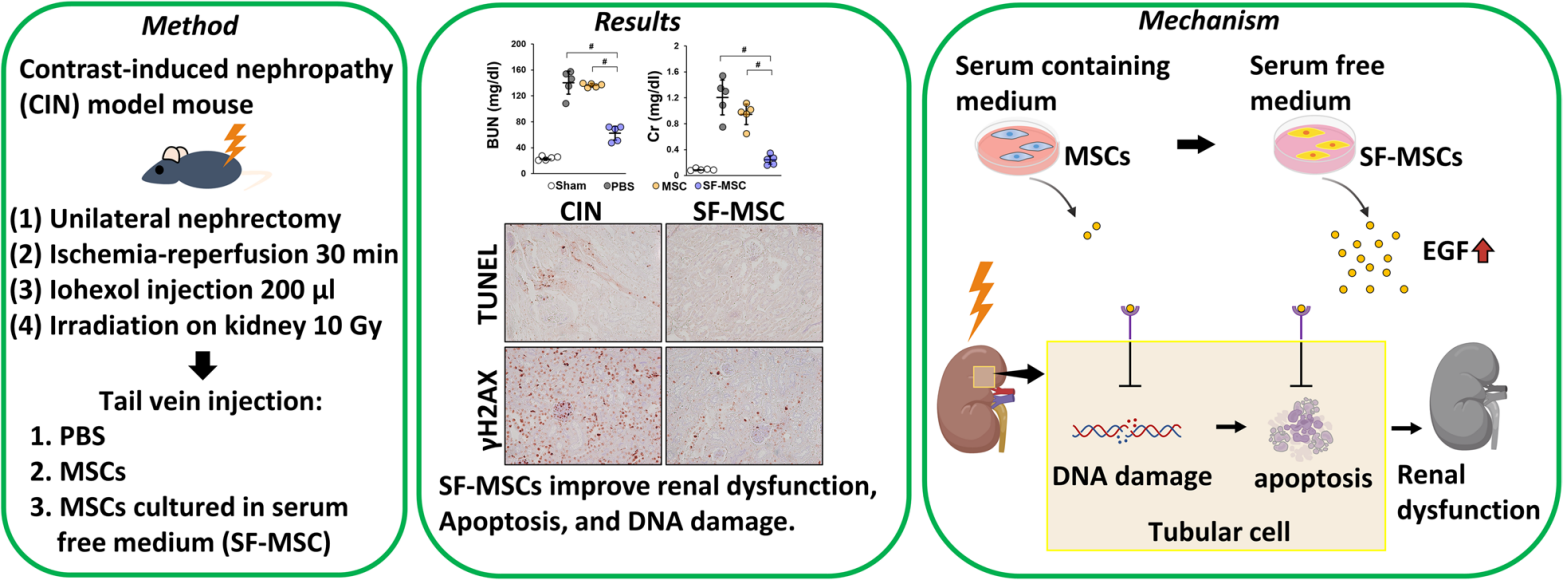

**Supplementary Information:**

The online version contains supplementary material available at 10.1186/s13287-023-03553-8.

## Background

Contrast media are used in various radiological diagnostic and therapeutic applications. However, acute contrast-induced nephropathy (CIN) is a serious side effect of the administration of contrast media. CIN is the third most common cause of acute renal failure in hospital onset, accounting for approximately 11% of all cases [1]. CIN patients have an increased risk of developing in-hospital complications, which include diseases with mortality rates of 10–20% and occasionally develop chronic renal failure [2]. The in-hospital mortality rate of patients with CIN is approximately five times that of patients who received contrast media but did not develop CIN, and the 1- and 5-year mortality rates of patients with CIN are approximately four times higher than those of patients without CIN [2]. CIN is defined as either a 0.5 mg/dL increase in the absolute serum creatinine value or a 25% increase in serum creatinine from baseline within 48–72 h after administration of contrast media [3]. The administration of saline before and after contrast media administration is the only preventative strategy for CIN in a clinical setting and effective treatments are lacking.

The pathological mechanism of CIN remains mostly poorly understood. However, previous studies have shown that the pathogenesis of CIN involves reactive oxygen stress, ischemic injury of renal arteries, and renal cell apoptosis [4]. Several studies have indicated that suppressing renal cell apoptosis improves CIN [5–7]. Therefore, strategies to target the apoptosis of renal cells may represent a potential therapeutic approach for CIN.

Mesenchymal stem cells (MSCs) are multi-potent adult stem cells that are isolated from adipose tissue, bone marrow, umbilical cord blood, and placenta and have anti-inflammatory, anti-fibrotic, and anti-apoptotic effects [8–11]. Previous studies have demonstrated that the administration of MSCs exerts beneficial effects on various diseases in animal models, and their anti-inflammatory and anti-fibrotic effects are enhanced by preconditioning with various factors, including hypoxia [12, 13], cytokines [14, 15], and pharmacological compounds [16, 17]. Our recent studies showed that culturing MSCs in serum-free medium enhances their anti-inflammatory and anti-fibrotic effects [18, 19]. In clinical applications, using serum-free medium for culturing MSCs has many advantages, such as absence of the need to check for differences in serum lots, shorter culture period, more efficient and stable cell proliferation, and reduced risk of infection from serum-derived components among others. However, whether serum-free culturing potentiates the anti-apoptotic effects of MSCs is unknown.

In this study, we investigated whether the administration of MSCs cultured in serum-free medium (SF-MSCs) improved the renal function of CIN model mice.

## Methods

### Preparation of MSCs

MSCs were derived from adipose tissues obtained from patients who underwent breast reconstruction. MSCs were cultured in Dulbecco’s modified Eagle’s medium (DMEM; Sigma-Aldrich, St. Louis, MO, USA) with 10% fetal bovine serum (FBS) (Sigma-Aldrich) or STK (Kanto Reagents, Tokyo, Japan). Cells up to passage 4 were used in all experiments. The Medical Ethics Committee of Hiroshima Graduate School of Biomedical Science permitted the collection of adipose tissue (permit number: E-1516, registered on January 29, 2019). All patients provided written informed consent.

### Animals

Eight-week-old male C57BL/6 mice were purchased from Charles River Laboratories Japan (Yokohama, Japan). A total of 45 mice were used in this study. All mice were reared in standard cages under a 12-h light–dark cycle at approximately 25 °C and 40–60% humidity, and were provided with free access to food and water at the Laboratory Animal Center of Hiroshima University (Hiroshima, Japan). All experimental procedures were approved by the Institutional Animal Care and Use Committee of Hiroshima University (Hiroshima, Japan) (permit number: A22-12) and performed in accordance with the “Guide for the Care and Use of Laboratory Animals, 8th ed, 2010” (National Institutes of Health, Bethesda, MD, USA). The study results were reported in accordance with ARRIVE guidelines 2.0.

### Experimental protocol

CIN model mice were established as previously described [20]. Briefly, mice were anesthetized with a mixture of medetomidine (0.3 mg/kg; Kyoritsuseiyaku, Tokyo, Japan), midazolam (4.0 mg/kg; Sand, Tokyo, Japan), and butorphanol (5.0 mg/kg; Meiji Seika Pharma, Tokyo, Japan). The skin on the right side of the spine was incised to expose the right kidney; the right kidney was removed, and the incision was closed with sutures. One week after the procedure, mice were anesthetized, and the left kidney was exposed. The left renal artery was clamped for 30 min to induce ischemia–reperfusion injury, and 200 µL of iohexol (Omnipaque, GE Healthcare, Tokyo, Japan) was injected using a retro-orbital injection method, as described previously [21]. The left kidney received 10 Gy of irradiation in an X-ray generator (MBR1520R-3, Hitachi Power Solutions, Ibaraki, Japan). The entire body, except for the left kidney, was shielded from X ray by a lead plate. After irradiation, MSCs (1.0 × 10^5^ cells/mouse) in 100 µL of phosphate-buffered saline (PBS) were immediately injected through the tail vein. In one experiment, mice were randomized into three treatment groups (n = 5 in each group): PBS, MSC, SF-MSC groups. In a second experiment, mice were randomized into three treatment groups (n = 5 in each group): PBS, NC siRNA SF-MSC, EGF siRNA SF-MSC. In a supplemental experiment, 250 µg/kg recombinant human EGF (rhEGF) (R&D Systems; 236-EG-01M) in 100 µL PBS was injected using the same method, and then mice were randomized into two groups (n = 5 in each group): PBS and rhEGF. The incision was then closed with sutures. At 24 h after tail vein injection, mice were sacrificed by exsanguination under anesthesia (medetomidine 0.3 mg/kg, midazolam 4.0 mg/kg, and butorphanol 5.0 mg/kg intraperitoneally) and kidneys and blood were collected.

### Histological analysis

Hematoxylin–eosin (HE) and immunohistochemical staining were performed using established methods [20, 22, 23]. Renal tissue samples were formalin-fixed and paraffin-embedded. Sections (2 µm thick) were prepared for HE staining. HE-stained sections were used to estimate tubular injury, as described previously [24]. Ten high magnification fields (×200) of the renal cortex and cortico-medullary junction were randomly selected from each mouse. The extent of degeneration and detachment of tubular cells was scored as follows: 0, normal; 1, involvement of 1–25% of cells; 2, involvement of 26–50%; 3, involvement of 51–75%; and 4, involvement of 76–100%.

Sections (4 µm thick) were also prepared for TdT-mediated dUTP nick end labeling (TUNEL) and γH2AX staining. TUNEL staining was performed using an in situ Apoptosis Detection kit (MK500; Takara, Shiga, Japan), following the manufacturer’s protocol for paraffin-embedded sections. For γH2AX staining, rabbit monoclonal anti-phospho-histone H2AX antibody (1:500; Cell Signaling Technology) was used as primary antibody. The number of TUNEL-positive cells and γH2AX-positive cells were counted in 10 randomly selected fields (at magnification × 200) in the corticomedullary differentiation area, and data were analyzed using ImageJ software.

### Western blotting

Western blot analysis was performed on kidney lysates and cultured cell extracts as previously described [25]. The following primary antibodies were used for analysis: rabbit polyclonal anti-phospho-H2AX (9718S; Cell Signaling Technology), rabbit polyclonal anti-cleaved caspase-3 antibody (9664S; Cell Signaling Technology), rabbit polyclonal anti-cleaved PARP (5625 T; Cell Signaling Technology), mouse monoclonal anti-GAPDH antibody (Sigma-Aldrich), and mouse monoclonal anti-α-tubulin antibody (Sigma-Aldrich). The intensity of each band was analyzed using ImageJ software and standardized by the level of either GAPDH or α-tubulin.

### Preparation of conditioned medium

To generate conditioned medium from MSCs, cells were seeded in 10-cm dishes and cultured in STK or DMEM containing 10% FBS. Once the cells achieved 70% confluence, the medium was replaced with DMEM containing 0.1% FBS, and the cells were cultured for 48 h. The medium was collected (indicated as SF-MSC-CM and MSC-CM) and used for further experiments. HK-2 cells were cultured in DMEM containing 5% FBS. Conditioned medium was generated using same method as the control for ELISA analysis.

### Irradiation experiment

HEK-293 cells were obtained from the American Type Culture Collection (ATCC, Manassas, VA, USA). Cells were cultured in Dulbecco’s Modified Eagle Medium (high glucose) (Nacalai Tesque, Kyoto, Japan) containing 10% FBS (Nichirei Bioscience, Tokyo, Japan), and penicillin/streptomycin (Nacalai Tesque).

HEK-293 cells were cultured in SF-MSC-CM, MSC-CM, or DMEM containing 0.1% FBS for 24 h and then exposed to 30 Gy X-ray irradiation (MBR1520R-3, Hitachi Power Solutions, Ibaraki, Japan). After 24 h, HEK-293 cells were collected for further analysis.

Erlotinib hydrochloride (FUJIFILM Wako Pure Chemical Corporation, Osaka, Japan) was used to inhibit EGFR signaling in HEK-293 cells. When the medium of HEK-293 cell cultures was replaced with SF-MSC-CM, erlotinib was added at a concentration of 10 μM.

### Enzyme-linked immunosorbent assay (ELISA)

ELISA kits for vascular endothelial growth factor (VEGF), hepatocyte growth factor (HGF), milk fat globule-EGF factor 8 (MFGE8), epidermal growth factor (EGF), heparin-binding EGF-like growth factor (HB-EGF), and transforming growth factor-alpha (TGF-α) were obtained from R&D Systems. Assays were performed following the manufacturer’s protocols. Concentrations were normalized to the total protein content or the number of cells.

### siRNA transfection

SF-MSCs were transfected with 20 nM siRNA against EGF (siRNA ID 146221; Applied Biosystems) or negative control siRNA (Applied Biosystems) using Lipofectamine 2000 Transfection Reagent (Thermo Fisher Scientific). For in vivo experiments, after 24 h transfection, the cells were collected and administered to mice.

### Quantitative real‐time reverse transcription PCR

RNA extraction and real-time reverse transcription PCR were performed following the previously described methods [14]. Specific oligonucleotide primers and probes for human EGF (assay ID: Hs01099990_m1) and 18S rRNA (endogenous control) were obtained as TaqMan Gene Expression Assays (Applied Biosystems, Foster City, CA, USA). mRNA levels were normalized to the level of 18S rRNA.

### Statistical analysis

All data are expressed as means ± standard deviations (S.D.). For multiple group comparisons, one-way ANOVA followed by Tukey–Kramer’s post-hoc test was applied. Comparisons between two groups were analyzed by Student’s t-test. *P* < 0.05 was considered statistically significant.

## Results

### Renoprotective effects of MSCs and SF-MSCs in CIN model mice

**Fig. 1:**
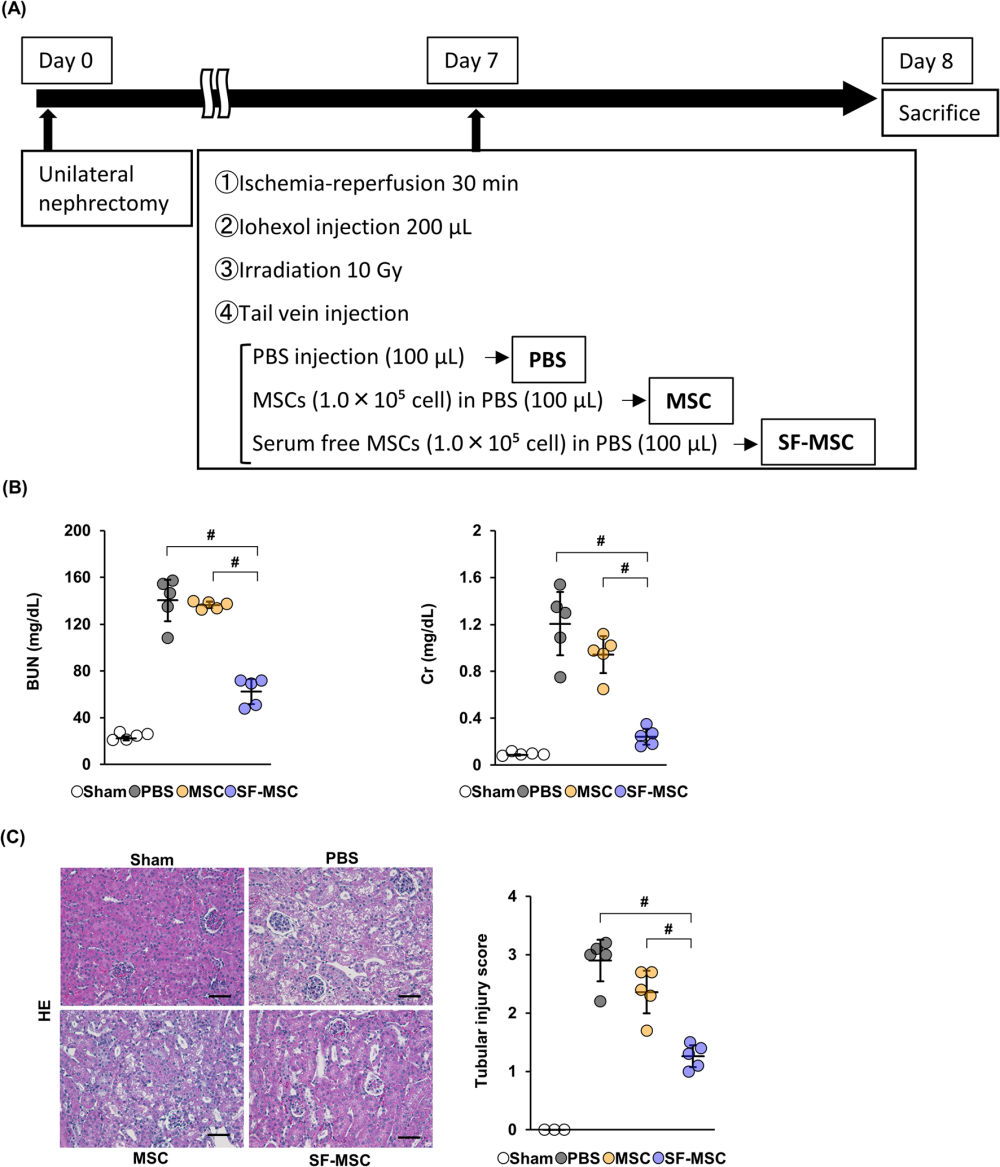
Therapeutic effects of SF-MSCs on CIN model mice. **A** Establishment of CIN model mice and treatment groups. CIN model mice were divided into three groups after irradiation: PBS: PBS injection (100 µL); MSC: MSCs injection (1.0 × 10^5^ cells in 100 µL PBS); and SF-MSC: SF-MSCs injection (1.0 × 10^5^ cells in 100 µL PBS). **B** Serum levels of blood urea nitrogen (BUN) and creatinine (Cr) (n = 5 in each group). **C** Representative hematoxylin–eosin (HE) staining images and tubular injury score of the kidney. Ten high magnification fields (×200) of the renal cortex and cortico-medullary junction were randomly selected from each mouse (n = 3 in sham, n = 5 in other groups). The extent of degeneration and detachment of tubular cells were scored as described in Methods. All assessments were done by two investigators blinded to experimental conditions. Scale bar: 100 µm. Data are means ± S.D. ^#^*P* < 0.01 (one-way ANOVA followed by Tukey–Kramer’s post-hoc test)

To investigate the potential therapeutic effect of SF-MSCs on CIN, we established CIN model mice and treated mice with PBS (PBS group), MSCs (MSC group), or SF-MSCs (SF-MSC group), as described in Methods (Fig. 1A). We first examined the serum levels of blood urea nitrogen (BUN) and creatinine (Cr) in CIN model mice and found that the levels of BUN and Cr were elevated in the PBS group (Fig. 1B). While there was no difference in levels between the PBS and MSC group, the levels were significantly reduced in the SF-MSC group.

In histological analysis, HE staining of kidney in the PBS group showed swollen tubular cells with vacuoles (a pathological feature of osmotic nephrosis) and an increase in the tubular injury score (Fig. 1C), consistent with previous reports [23]. There was no difference in tubular injury scores between the PBS and MSC groups; however, the score was significantly decreased in the SF-MSC group. Together, these findings demonstrate that administration of SF-MSCs suppressed tubular damage and improved renal function in CIN model mice.

### Effects of MSCs and SF-MSCs on DNA damage in the CIN model mouse kidney

**Fig. 2:**
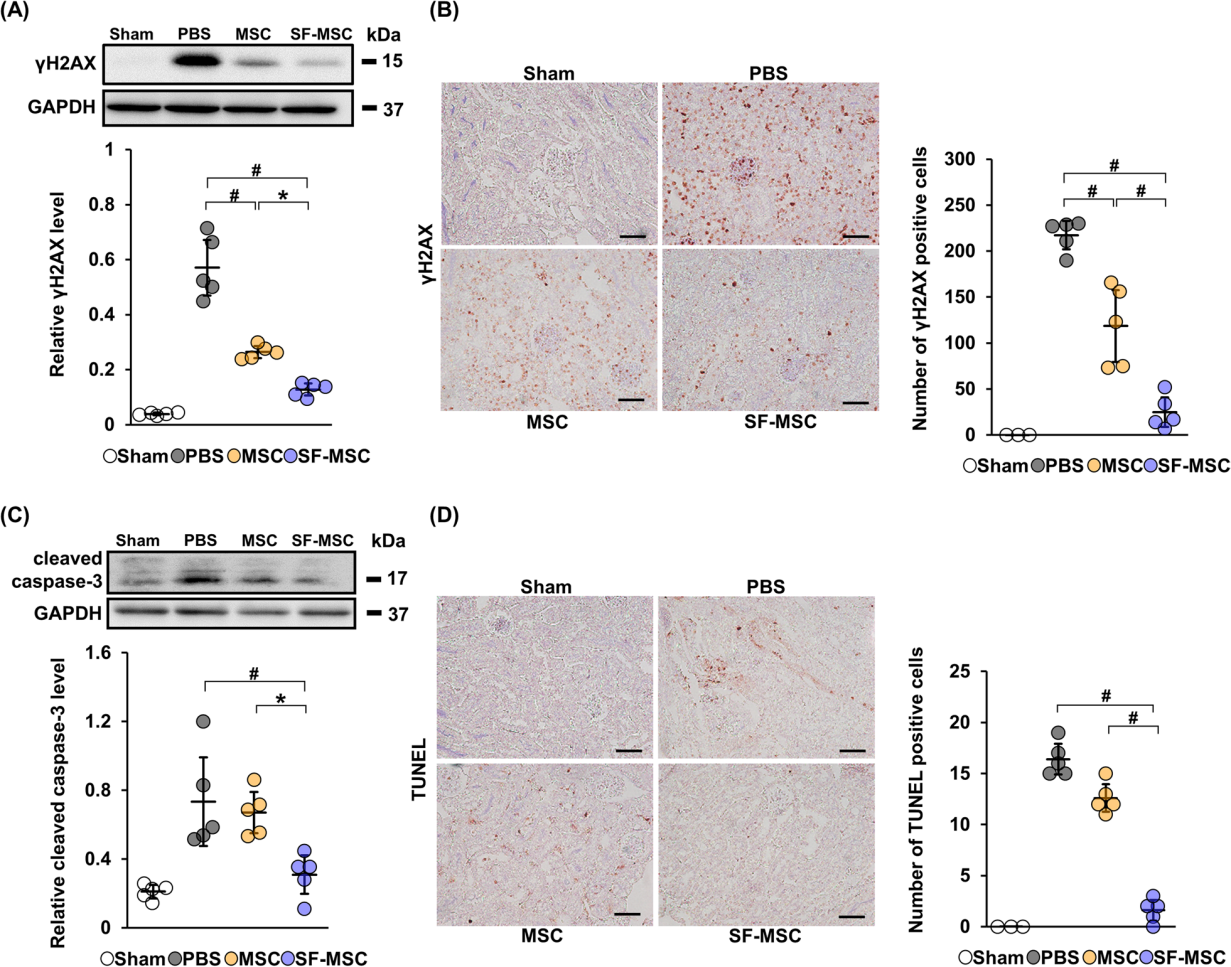
Effects of MSCs and SF-MSCs on DNA damage and apoptosis in the CIN model mouse kidney. **A** Immunoblotting analysis of γH2AX in kidney tissue from CIN model mice sacrificed at 24 h after tail vein injection (n = 5 in each group). GAPDH was used as a loading control. Full-length blots are presented in Additional file 1: Fig. 1a. **B** Representative immunostaining images for γH2AX in the kidney. Scale bar: 100 µm. Graph shows the average number of γH2AX-positive cells (n = 3 in sham, n = 5 in other groups). Ten high magnification fields (×200) of the renal cortex and cortico-medullary junction were randomly selected, and the average number of γH2AX-positive cells per field was calculated. **C** Immunoblotting analysis of cleaved caspase-3 in kidney tissue from CIN model mice sacrificed at 24 h after tail vein injection (n = 5 in each group). GAPDH was used as a loading control. Full-length blots are presented in Additional file 2: Fig. 1b. **D** Representative images for TdT-mediated dUTP nick end labeling (TUNEL) staining in the kidney. Scale bar: 100 µm. Graph shows the average number of TUNEL-positive cells (n = 3 in sham, n = 5 in other groups). Data are means ± S.D. ^#^*P* < 0.01, *P < 0.05 (one-way ANOVA followed by Tukey–Kramer’s post-hoc test)

Our previous study demonstrated that kidney injury in CIN model mice was enhanced by an increase in DNA damage [20]. To investigate whether SF-MSCs improve DNA damage in the kidney of CIN model mice, we evaluated the expression of γH2AX, a marker of chromosomal damage, using immunoblotting and immunohistochemistry. We found that the level of γH2AX was remarkably increased in the PBS group, and this increase was significantly suppressed in the MSC group (Fig. 2A, Additional file 1A: Fig. S1a). Further suppression was observed in the SF-MSC group. A similar trend was observed in the immunohistochemical staining analysis of γH2AX-positive cells (Fig. 2B). Taken together, these results indicate that MSCs attenuate the DNA damage in kidney tissue of CIN model mice, and the therapeutic effect was enhanced by culturing MSCs in serum-free medium.

### Effects of MSCs and SF-MSCs on apoptosis in the CIN model mouse kidney

Previous studies have shown that renal tubular cell apoptosis plays a role in CIN [4]. Therefore, we investigated the effects of SF-MSCs on tubular cell apoptosis in CIN model mice. First, we evaluated the expression of cleaved caspase-3, an effector caspase of apoptosis [26], in kidney tissue. We found that the protein level of cleaved caspase-3 was elevated in the PBS group (Fig. 2C, Additional file 1A: Fig. S1b). While the protein level of cleaved caspase-3 was not statistically different between PBS and MSC groups, cleaved caspase-3 level was markedly suppressed in the SF-MSC group. Next, we performed immunohistochemical staining analysis to evaluate tubular cell apoptosis. The PBS group showed an increase in the number of TUNEL-positive cells (Fig. 2D). Whereas there was no difference in TUNEL-positive cells between PBS and MSC groups, the number was significantly decreased in the SF-MSC group. These results indicate that SF-MSCs attenuate apoptosis in CIN model mice possibly.

### Strong suppression of apoptosis by conditioned medium from SF-MSCs

**Fig. 3:**
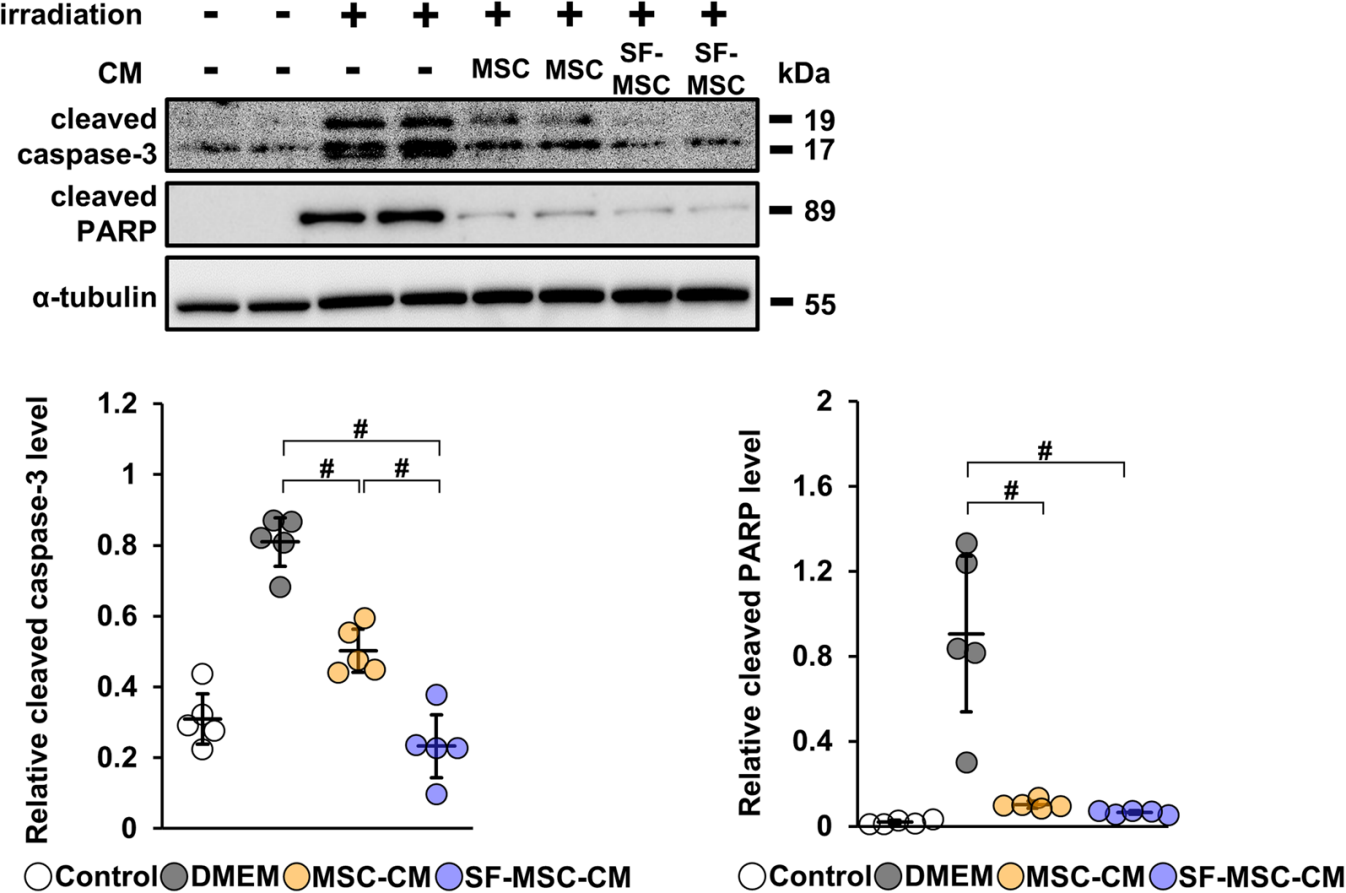
Conditioned medium from SF-MSCs strongly suppresses apoptosis of HEK-293 cells induced by irradiation. Immunoblotting analysis of cleaved caspase-3 and cleaved PARP in irradiated HEK-293 cells incubated for 24 h with conditioned medium from MSCs (MSC-CM) or SF-MSCs (SF-MSC-CM) (n = 5 in each group). α-Tubulin was used as a loading control. Full-length blots are presented in Additional file 3: Fig. 1c. Data are means ± S.D. ^#^*P* < 0.01 (one-way ANOVA followed by Tukey–Kramer’s post-hoc test)

We next investigated the apoptosis suppression mechanism of MSCs. Previous studies have demonstrated that SF-MSCs enhance their anti-fibrotic activity in a paracrine manner [18, 19]. We thus explored whether SF-MSCs enhance their anti-apoptotic effects in a paracrine manner. We incubated HEK-293 cells for 24 h in SF-MSC-CM (SF-MSC-CM group), MSC-CM (MSC-CM group), or DMEM containing 0.1% FBS (DMEM group). The cells were then stimulated by irradiation to induce apoptosis; after 24 h, immunoblotting analysis was performed for cleaved caspase-3 and cleaved PARP, effectors of apoptosis [27]. The protein level of cleaved caspase-3 was greatly increased in the DMEM group (Fig. 3, Additional file 1B: Fig. S1c). Treatment with MSC-CM reduced cleaved caspase-3 levels, and further inhibition was observed in the SF-MSC-CM group. The protein level of cleaved PARP was also upregulated in the DMEM group, and this upregulation was significantly reduced in both the MSC-CM and SF-MSC-CM groups (Fig. 3, Additional file 1B: Fig. S1c). These results indicate that conditioned medium from SF-MSCs exhibits anti-apoptotic effects in a paracrine manner.

### Increased EGF secretion from MSCs cultured in serum-free medium

**Fig. 4:**
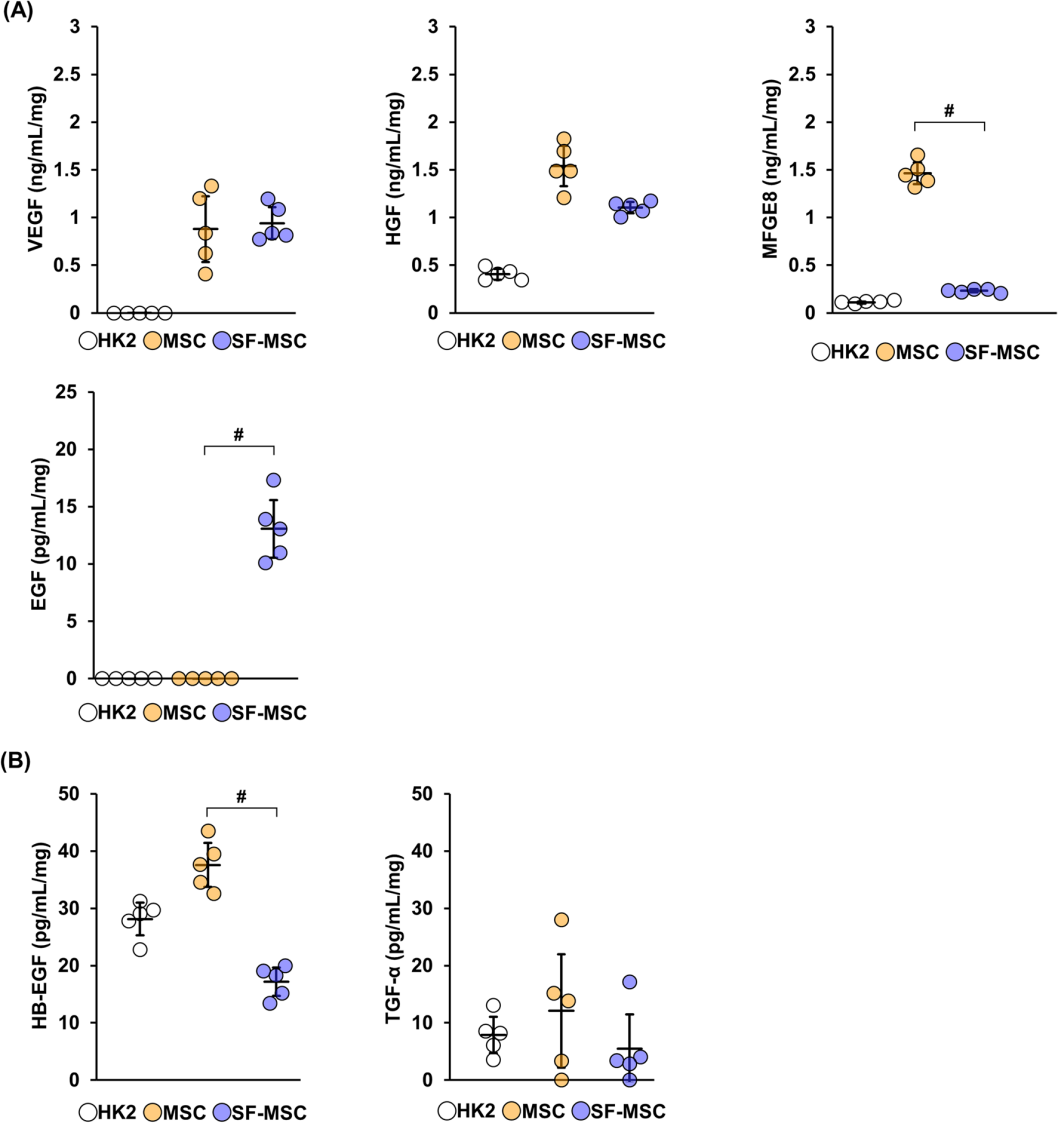
Culturing MSCs in serum-free medium increases the secretion of EGF. MSCs, SF-MSCs, and HK-2 cells were cultured in DMEM containing 0.1% FBS for 48 h, and then the culture supernatants were collected as conditioned medium. **A** The concentrations of vascular endothelial growth factor (VEGF), hepatocyte growth factor (HGF), milk fat globule-EGF factor 8 (MFGE8), and epidermal growth factor (EGF) in each conditioned medium were measured by ELISA (n = 5 in each group). **B** The concentrations of heparin-binding EGF-like growth factor (HB-EGF) and transforming growth factor-alpha (TGF-α) in each conditioned medium were measured by ELISA (n = 5 in each group). Concentrations were normalized to the total protein content. Data are means ± S.D. ^#^*P* < 0.01 (one-way ANOVA followed by Tukey–Kramer’s post-hoc test)

Our results indicated that SF-MSCs enhance their anti-apoptotic effects in a paracrine manner. We next investigated whether the secretions of VEGF, HGF, MFGE-8, and EGF, inhibitors of apoptosis [28–33], were enhanced in serum-free culture conditions. We compared the concentration of these factors in CM from MSCs, SF-MSCs, and HK-2 cells using ELISA. Among all investigated factors, only EGF was significantly and specifically increased only in SF-MSC-CM but not in MSC-CM (Fig. 4A). Additionally, similar results were obtained when the EGF concentration was normalized to the number of cells instead of the total protein content (Additional file 3: Fig. S3).

We then examined the concentrations of TGF-α and HB-EGF, which bind to the EGF receptor and share common activity pathway with EGF [34]. TGF-α and HB-EGF levels were not increased in SF-MSC-CM compared with those in MSC-CM (Fig. 4B). Taken together, these results suggest that the anti-apoptotic effect of SF-MSCs may involve the increased secretion of EGF.

### Effects of EGF knockdown in SF-MSCs on renal function

**Fig. 5:**
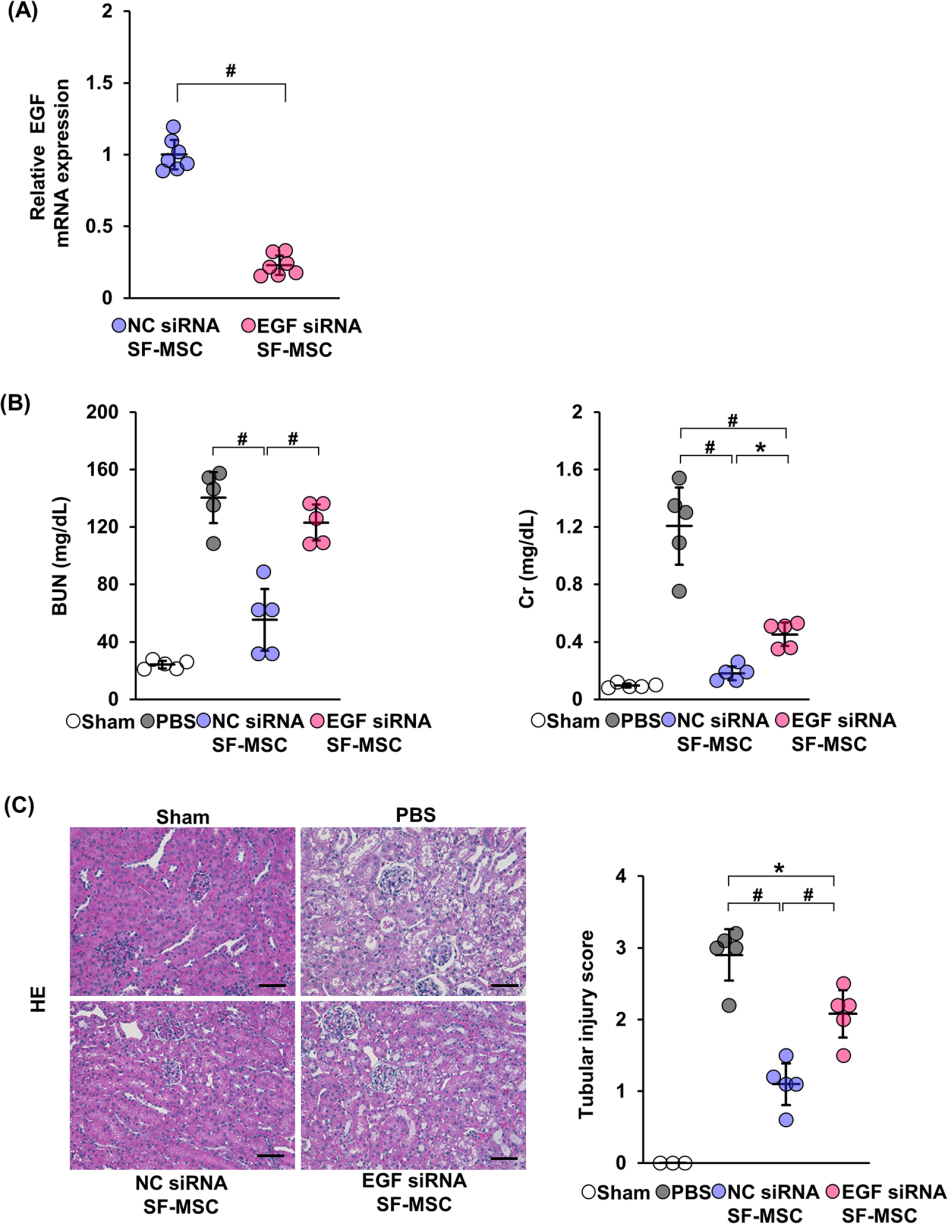
Knockdown of EGF in SF-MSC reduces the renoprotective effects in CIN model mice. **A** Graph showing the knockdown efficiency of EGF siRNA in SF-MSCs (n = 7 in each group). **B** Serum levels of BUN and Cr (n = 5 in each group). **C** Representative hematoxylin–eosin (HE) staining image and tubular injury score of the kidney (n = 3 in sham, n = 5 in other groups). Ten high magnification fields (×200) of the renal cortex and cortico-medullary junction were randomly selected from each mouse. The extent of degeneration and detachment of tubular cells was scored. Scale bar: 100 µm. Data are means ± S.D. ^#^*P* < 0.01, *P < 0.05 (one-way ANOVA followed by Tukey–Kramer’s post-hoc test)

We hypothesized that EGF plays an important role in the protection of renal function by SF-MSCs in CIN model mice. To determine whether EGF secreted by SF-MSCs protects renal function, we transfected SF-MSCs with EGF-siRNA (EGF siRNA SF-MSCs) or negative control siRNA SF-MSCs (NC siRNA SF-MSCs). First, we confirmed successful knockdown of EGF in EGF-siRNA SF-MSCs (Fig. 5A). We next examined the serum levels of BUN and Cr in CIN model mice injected with EGF siRNA SF-MSCs or NC siRNA SF-MSCs (EGF siRNA SF-MSC group and NC siRNA SF-MSC group, respectively). The result showed that BUN and Cr were significantly elevated in the EGF siRNA SF-MSC group compared to the NC siRNA SF-MSC group (Fig. 5B). Similar results were observed in the tubular injury score analysis (Fig. 5C).

### Effects of EGF knockdown in SF-MSCs on DNA damage and apoptosis

**Fig. 6:**
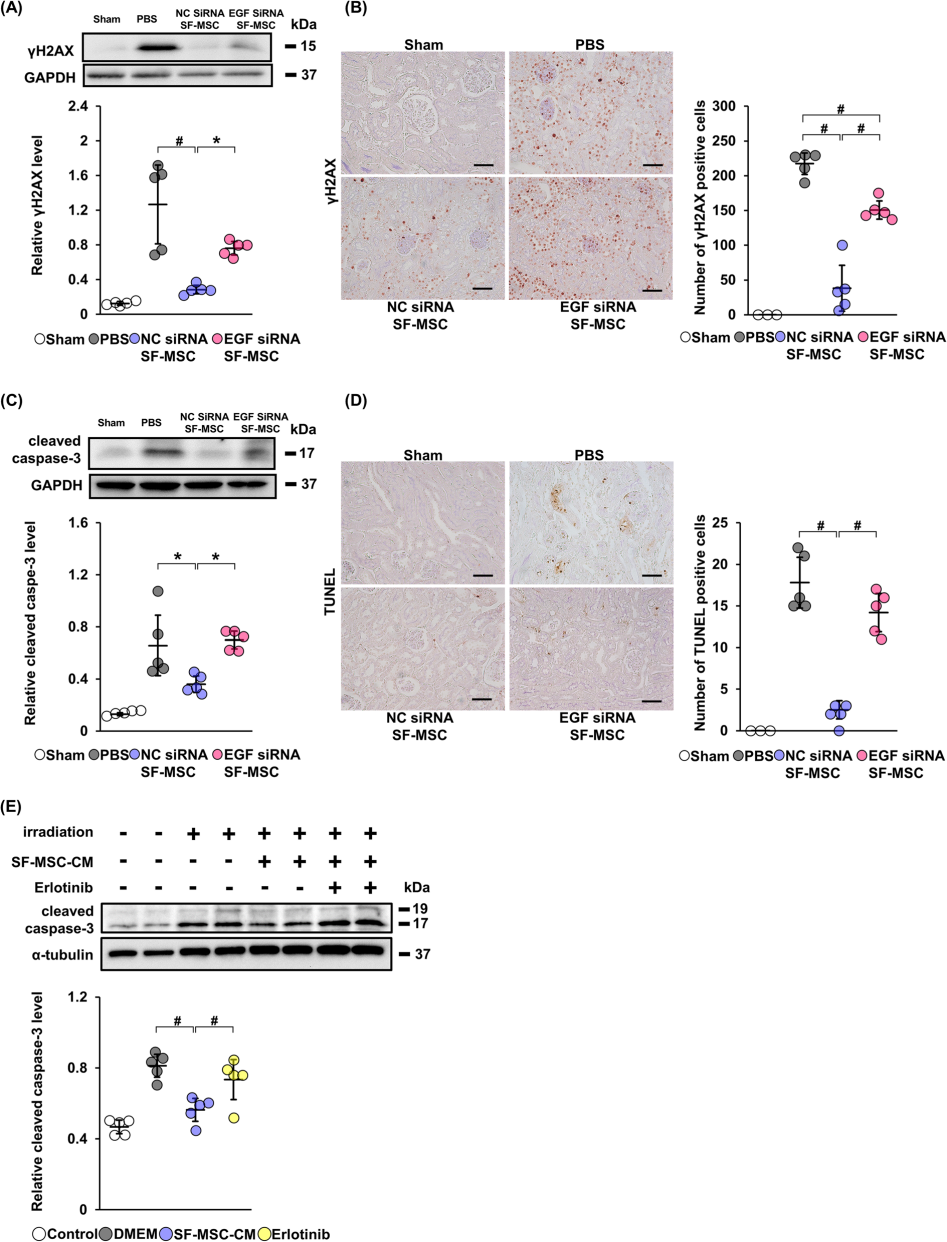
Knockdown of EGF in SF-MSCs reduces the anti-apoptotic effect in CIN model mice. **A** Immunoblotting analysis of γH2AX in kidney tissue from CIN model mice sacrificed at 24 h after tail vein injection (n = 5 in each group). GAPDH was used as a loading control. Full-length blots are presented in Additional file 1: Fig. 1d. **B** Representative immunostaining of γH2AX in kidney from each group of mice. Scale bar: 100 µm. Graph shows the average number of γH2AX-positive cells (n = 3 in sham, n = 5 in other groups). Ten high magnification fields (×200) of the renal cortex and cortico-medullary junction were randomly selected, and the average number of γH2AX positive cells per field was calculated. **C** Immunoblotting analysis of cleaved caspase-3 in kidney tissue from CIN model mice sacrificed at 24 h after tail vein injection. GAPDH was used as a loading control (n = 5 in each group). Full-length blots are presented in Additional file 1: Fig. 1e. **D** Representative image of TdT-mediated dUTP nick end labeling (TUNEL) staining in the kidney from each group of mice. Scale bar: 100 µm. Graph shows the average number of TUNEL-positive cells (n = 3 in sham, n = 5 in other groups). Ten high magnification fields (×200) of the renal cortex and cortico-medullary junction were randomly selected, and the average number of TUNEL positive cells per 1 field was calculated. **E** Immunoblotting analysis of cleaved caspase-3 in irradiated HEK-293 cells incubated for 24 h with SF-MSC-CM or SF-MSC-CM with 10 µM erlotinib (n = 5 in each group). α-Tubulin was used as a loading control. Full length blots are presented in Additional file 1: Fig. 1f. Data are means ± S.D. ^#^*P* < 0.01, *P < 0.05 (one-way ANOVA followed by Tukey–Kramer’s post-hoc test)

We next investigated the effects of knockdown of EGF in SF-MSCs on DNA damage and apoptosis in the kidney of CIN model mice. Immunoblotting analysis showed that the level of γH2AX in the NC siRNA SF-MSC group was reduced more significantly than that in the EGF siRNA SF-MSC group (Fig. 6A, Additional file 1C: Fig. S1d). A similar trend was observed in immunohistochemical analysis of γH2AX (Fig. 6B). Both the protein level of cleaved caspase-3 in kidney lysates and the number of TUNEL-positive cells in kidney tissues were higher in the EGF siRNA SF-MSC group compared with those in the NC siRNA SF-MSC group (Fig. 6C, D, Additional file 1C: Fig. S1e).

To confirm the role of EGF secreted from SF-MSCs in suppressing apoptosis, we treated irradiated HEK-293 cells with SF-MSC-CM and EGFR inhibitor erlotinib. In the SF-MSC-CM with erlotinib group, the protein level of cleaved caspase-3 was significantly increased compared with that in the SF-MSC-CM group (Fig. 6E, Additional file 1C: Fig. S1f). This result demonstrates that SF-MSC-CM suppress apoptosis via EGFR signaling.

### Effects of rhEGF administration in CIN model mice

Next, we investigated whether administration of rhEGF alone exerts renoprotective and anti-apoptotic effects in CIN model mice. We found that serum levels of BUN and Cr were significantly reduced in the rhEGF group compared with the PBS group (Additional file 2: Fig. S2a). A similar result was obtained in the tubular injury score analysis (Additional file 2: Fig. S2b). Both the protein level of cleaved caspase-3 in kidney lysates and the number of TUNEL-positive cells in kidney tissues were decreased in the rhEGF group compared with the PBS group (Additional file 2: Fig. S2c, S2d, Additional file 1D: Fig. S1g). Taken together, these results demonstrate that administration of rhEGF improves renal dysfunction and suppresses apoptosis in CIN model mice.

## Discussion

In this study, we demonstrated that SF-MSCs improve renal function and attenuate tubular cell apoptosis in CIN model mice. We also showed that apoptosis induced by irradiation was attenuated by the addition of conditioned medium from SF-MSCs in a paracrine manner. Knockdown of EGF by siRNA attenuated the renoprotective and anti-apoptotic effects of SF-MSCs on CIN model mice. These findings indicate that SF-MSCs exhibit anti-apoptotic activity and may represent an effective therapeutic strategy for CIN.

We recently reported that the combination of ionizing radiation and contrast medium induces DNA double-strand breaks in a CIN model [20]. Double-strand breaks cause cell dysfunction, cell death, and subsequent tissue damage [35]. In this study, we found that the level of γH2AX, a chromosomal damage marker, was markedly decreased in the kidney of CIN model mice treated with SF-MSCs, whereas γH2AX level was only slightly decreased by the treatment with MSCs. These findings were observed by both immunoblotting and immunohistochemical analysis of kidney sections. These results indicate that SF-MSCs markedly reduce DNA damage in CINs, which may prevent cell dysfunction and apoptosis induction. Indeed, administration of SF-MSCs strongly suppressed the expression of cleaved-caspase3 and the number of TUNEL-positive cells in CIN model mice. Collectively, these results demonstrate that treatment with SF-MSCs reduces DNA damage and suppresses apoptosis in CIN model mice, which in turn results in the survival of tubular cells and improves renal function and tubular damage scores.

In in vitro experiments, we found that SF-MSCs suppress apoptosis in a paracrine manner. We also found that the secretion of EGF was enhanced in SF-MSCs compared with that in MSCs. To our best knowledge, this is the first report that MSCs cultured in serum-free medium enhance EGF secretion. Binding of EGF to EGF receptor results in the promotion of cell growth and cell survival through several signaling pathways. The PI3K-AKT pathway is one of the major EGF receptor pathways that inhibits apoptosis by directly inhibiting proapoptotic proteins; this pathway inhibits FoxO, which regulates the transcription of proapoptotic proteins, and induces MDM2, which inhibits p53, an inducer of BAX [36]. Our findings showed that knockdown of EGF with siRNA attenuated the inhibitory effect of SF-MSCs on CIN-induced renal dysfunction and tubular apoptosis in CIN model mice. Furthermore, in the in vitro experiment using the EGFR inhibitor, we found that EGFR signaling played an essential role in suppressing apoptosis induced by SF-MSCs. These findings suggested that EGF secreted from SF-MSCs may inhibit apoptosis. Additionally, γH2AX was increased in SF-MSCs with EGF knockdown, and the improvement of DNA damage by SF-MSCs was attenuated. DNA damage and the subsequent activation of apoptosis also plays an important role in the progression of renal dysfunction [37, 38]. In this study, we found that culturing MSCs in serum-free medium increased the secretion of EGF and enhanced the anti-apoptotic effect of MSCs in CIN model mice. These results indicate that SF-MSCs may represent an effective treatment for CIN. We confirmed that administration of rhEGF improved renal function and suppressed apoptosis in CIN model mice. However, administration of EGF intravenously to primates has been reported to cause hypotension [39]. Therefore, it would be difficult to administer EGF alone to CIN patients in clinical situations.

There were some limitations in this study. In clinical situations, CIN is often observed in patients with chronic kidney disease, but a suitable animal model for this condition has not been established. Hence, we induced acute kidney injury in CIN model mice using a published method. Moreover, we could not demonstrate the mechanism by which EGF secretion from MSCs increased in serum-free medium. Further studies are required to elucidate this mechanism.

## Conclusions

This study demonstrates that MSCs cultured in serum-free medium have an enhanced ability to attenuate apoptosis in CIN model mice. Thus, SF-MSCs may represent an effective novel treatment for CIN.

### Supplementary Information

**Additional file 1A-D: Fig. S1.** Full-length western blot images. **a** Full-length blot images for Fig. 2A. **b** Full-length blot images for Fig. 2C. **c** Full-length blot images for Fig. 3. **d** Full-length blot images for Fig. 6A. **e** Full-length blot images for Fig. 6C. **f** Full-length blot images for Fig. 6E. **g** Full-length blot images for Supplemental figure 2C.

**Additional file 2: Fig. S2.**. Recombinant human EGF improves renal dysfunction and suppresses apoptosis in CIN model mice. (a) Serum levels of BUN and Cr (n = 5 in each group). (b) Representative hematoxylin–eosin (HE) staining image and tubular injury score of the kidney (n = 3 in sham, n = 5 in other groups). Ten high magnification fields (×200) of the renal cortex and corticomedullary junction were randomly selected from each mouse. (c) Immunoblotting analysis of cleaved caspase-3 in kidney tissue from CIN model mice sacrificed at 24 h after tail vein injection. GAPDH was used as a loading control (n = 5 in each group). Full length blots are presented in Additional file 1: Fig. 1g. (d) Representative image of TdT-mediated dUTP nick end labeling (TUNEL) staining in the kidney from each group of mice. Scale bar: 100 µm. Graph shows the average number of TUNEL-positive cells (n = 3 in sham, n = 5 in other groups). Ten high magnification fields (×200) of the renal cortex and corticomedullary junction were randomly selected, and the average number of TUNEL-positive cells per field was calculated. Data are means ± S.D. ^#^*P* < 0.01 (Student's t-test).

**Additional file 3: Fig. S3.** Culturing MSCs in serum-free medium increases the secretion of EGF. MSCs, SF-MSCs, and HK-2 cells were cultured in DMEM containing 0.1% FBS for 48 h, and then the culture supernatants were collected as conditioned medium. Epidermal growth factor (EGF) in each conditioned medium were measured by ELISA (n = 4 in each group). Concentrations were normalized to the total number of cells. Data are means ± S.D. *P* < 0.01 (one-way ANOVA followed by Tukey–Kramer’s post-hoc test).

## Data Availability

The data that support the findings of this study are available from the corresponding author upon reasonable request.

## Abbreviations

BUN: Blood urea nitrogen
CIN: Contrast-induced nephropathy
CM: Conditioned medium
Cr: Creatinine
DMEM: Dulbecco’s modified Eagle’s medium
DMEM group: HEK-293 cells incubated with DMEM containing 0.1% FBS
EGF: Epidermal growth factor
EGF siRNA SF-MSC group: CIN model mice injected with EGF siRNA SF-MSCs
EGF siRNA SF-MSCs: SF-MSCs transfected with EGF siRNA
ELISA: Enzyme-linked immunosorbent assay
FBS: Fetal bovine serum
HB-EGF: Heparin-binding EGF-like growth factor
HE: Hematoxylin and eosin
HGF: Hepatocyte growth factor
MFGE8: Milk fat globule-EGF factor 8
MSC: MSCs cultured in MSC CM: conditioned medium generated from MSCs
MSC-CM group: HEK-293 cells incubated with MSC-CM
MSC group: CIN model mice injected with MSCs
MSCs: Mesenchymal stem cells
NC siRNA SF-MSC group: CIN model mice injected with NC siRNA SF-MSCs
NC siRNA SF-MSCs: SF-MSCs transfected with negative control siRNA
PBS: Phosphate-buffered saline
PBS group: CIN model mice injected with PBS
rhEGF: Recombinant human EGF
SD: Sprague–Dawley
SF-MSC CM: Conditioned medium generated from SF-MSCs
SF-MSC-CM group: HEK-293 cells incubated with SF-MSC-CM
SF-MSC group: CIN model mice injected with SF-MSCs
SF-MSCs: MSCs cultured in serum-free medium
TGF-α: Transforming growth factor-alpha
TUNEL: TdT-mediated dUTP nick end labeling
VEGF: Vascular endothelial growth factor
