# Supplementary material for: Adipose-derived mesenchymal stem cells cultured in serum-free medium attenuate acute contrast-induced nephropathy by exerting anti-apoptotic effects
Source: Stem Cell Res Ther. 2023 Nov 22;14:337. doi: 10.1186/s13287-023-03553-8 (PMC10664307; doi:10.1186/s13287-023-03553-8)

Additional file 1A

a Full-length blot images for figure.2 (A)

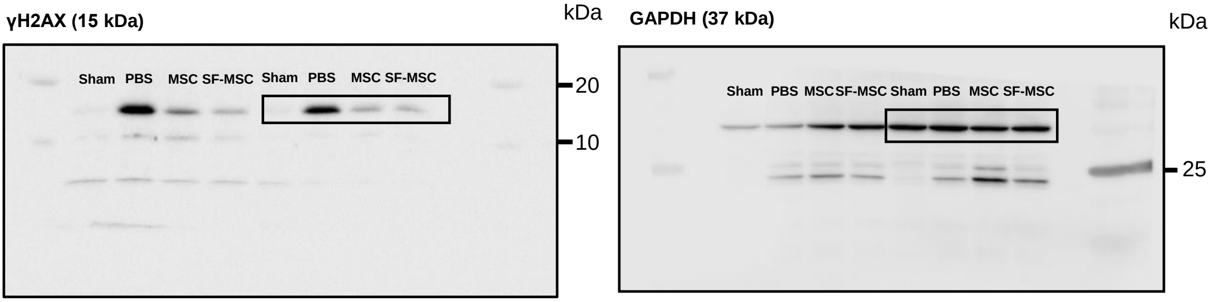

b Full-length blot images for figure.2 (C)

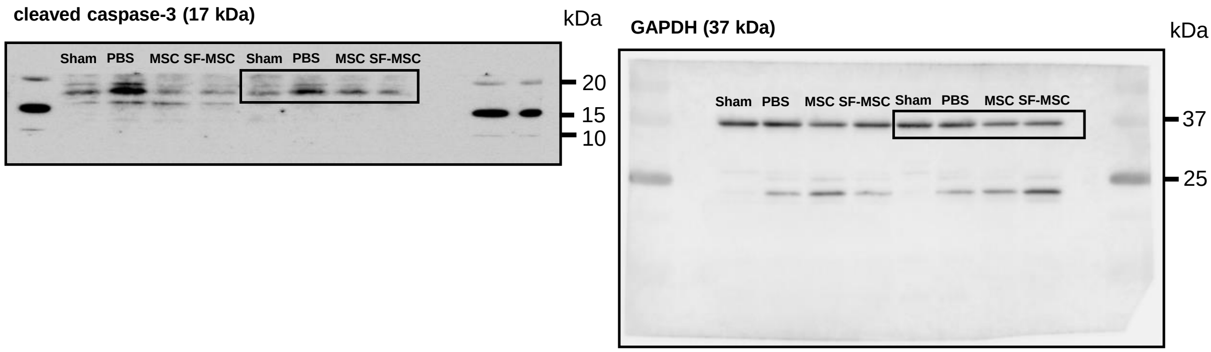

Additional file 1B

c Full-length blot images for figure.3

α-tubulin (55 kDa)

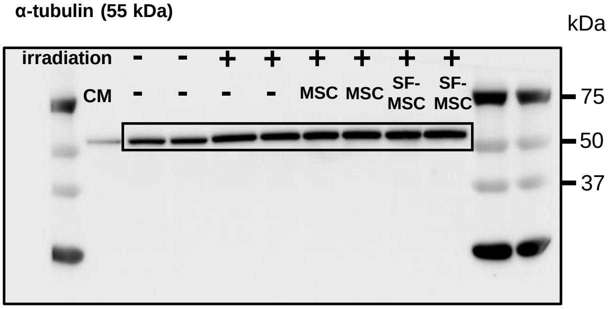

cleaved caspase-3 (37 kDa)

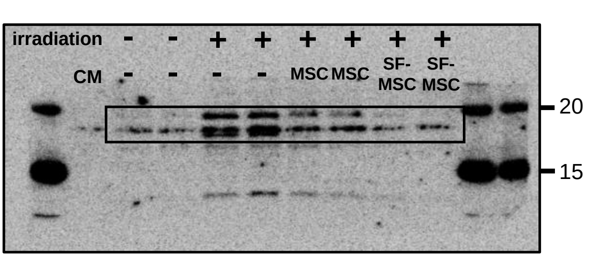

cleaved PARP (89 kDa)

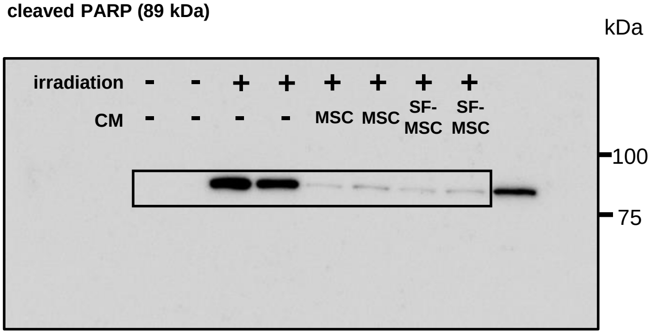

Additional file 1C

d Full-length blot images for figure.6 (A)

γH2AX (15 kDa) kDa

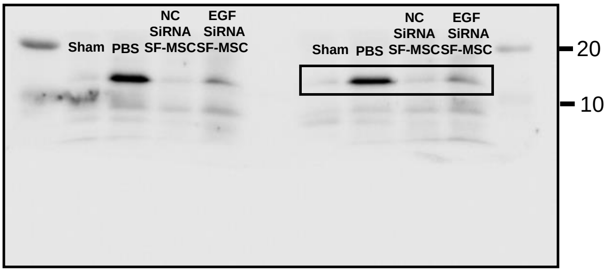

GAPDH (37 kDa) kDa

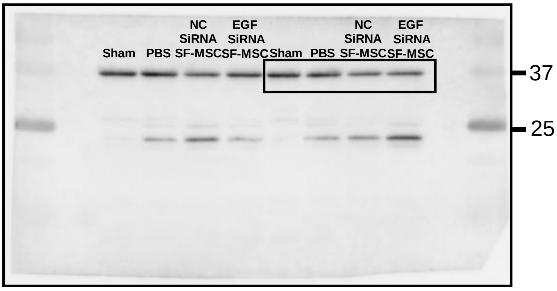

e Full-length blot images for figure.6 (C)

cleaver caspase-3 (17 kDa) kDa

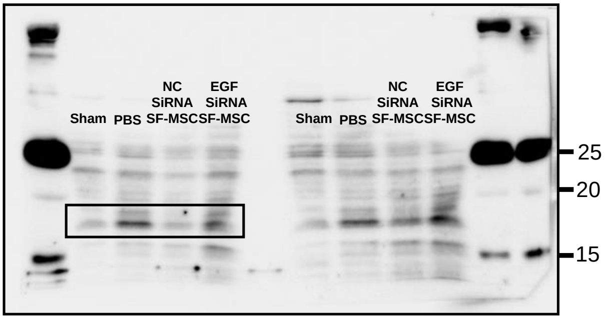

GAPDH (37 kDa) kDa

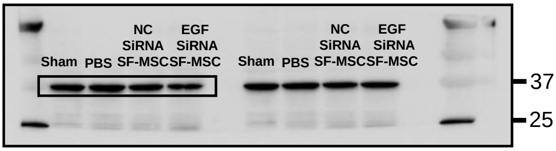

f Full-length blot images for figure.6 (E)

cleaver caspase-3 (17 kDa)

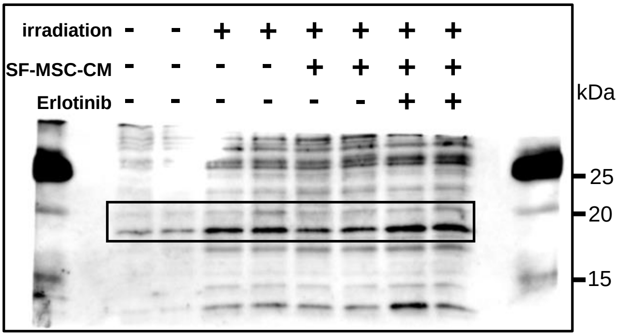

α-tubulin (55 kDa)

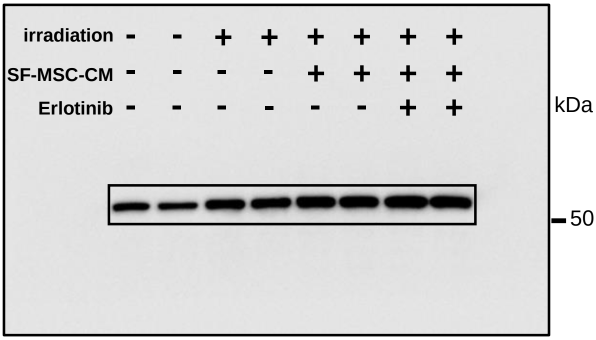

Additional file 1D

g Full-length blot images for Supplemental figure.2 (c)

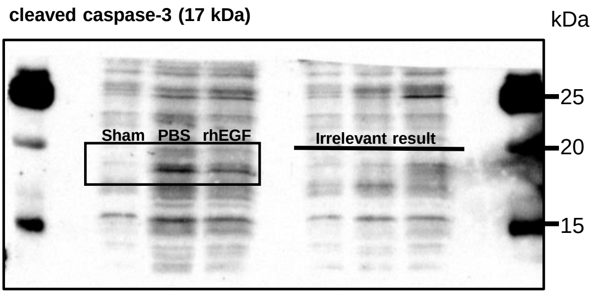

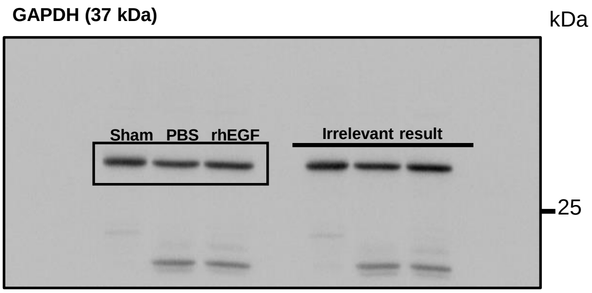

Supplement: Supplementary file 1 — Additional file 1A-D: Fig. S1. Full-length western blot images. a Full-length blot images for Fig. 2A. b Full-length blot images for Fig. 2C. c Full-length blot images for Fig. 3. d Full-length blot images for Fig. 6A. e Full-length blot images for Fig. 6C. f Full-length blot images for Fig. 6E. g Full-length blot images for Supplemental figure 2C. [file 13287_2023_3553_MOESM1_ESM.pdf]
