# Supplementary material for: Adipose-derived mesenchymal stem cells cultured in serum-free medium attenuate acute contrast-induced nephropathy by exerting anti-apoptotic effects
Source: Stem Cell Res Ther. 2023 Nov 22;14:337. doi: 10.1186/s13287-023-03553-8 (PMC10664307; doi:10.1186/s13287-023-03553-8)
Supplement: Supplementary file 2 — Additional file 2: Fig. S2.. Recombinant human EGF improves renal dysfunction and suppresses apoptosis in CIN model mice. (a) Serum levels of BUN and Cr (n = 5 in each group). (b) Representative hematoxylin–eosin (HE) staining image and tubular injury score of the kidney (n = 3 in sham, n = 5 in other groups). Ten high magnification fields (×200) of the renal cortex and corticomedullary junction were randomly selected from each mouse. (c) Immunoblotting analysis of cleaved caspase-3 in kidney tissue from CIN model mice sacrificed at 24 h after tail vein injection. GAPDH was used as a loading control (n = 5 in each group). Full length blots are presented in Additional file 1: Fig. 1g. (d) Representative image of TdT-mediated dUTP nick end labeling (TUNEL) staining in the kidney from each group of mice. Scale bar: 100 µm. Graph shows the average number of TUNEL-positive cells (n = 3 in sham, n = 5 in other groups). Ten high magnification fields (×200) of the renal cortex and corticomedullary junction were randomly selected, and the average number of TUNEL-positive cells per field was calculated. Data are means ± S.D. #P < 0.01 (Student's t-test). [file 13287_2023_3553_MOESM2_ESM.pdf]

Additional file 2: Supplemental Figure 2.

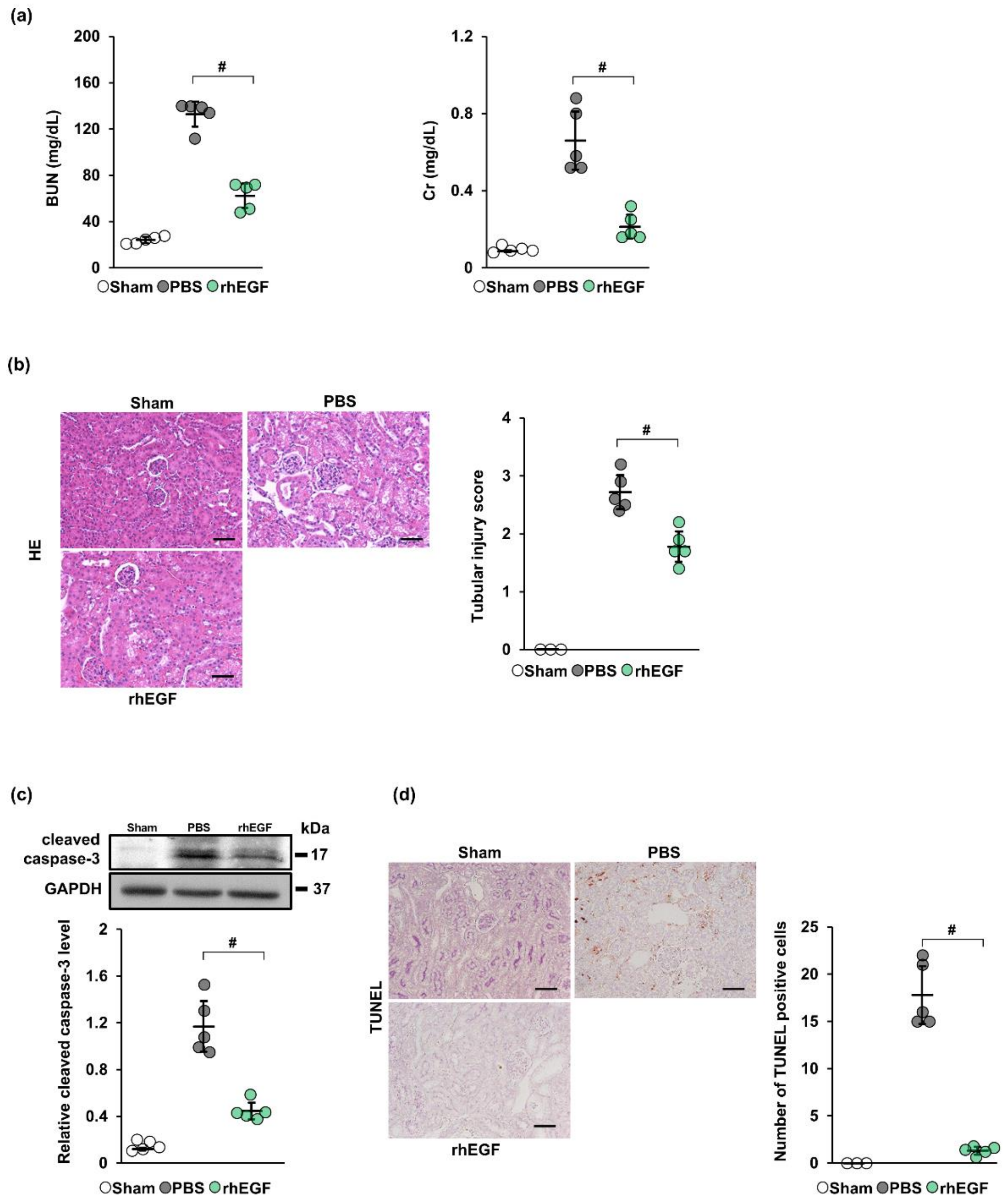

Supplemental Figure S2. Recombinant human EGF improves renal dysfunction and suppresses apoptosis in CIN model mice.

(a) Serum levels of BUN and Cr (n = 5 in each group). (b) Representative hematoxylin-eosin (HE) staining image and tubular injury score of the kidney (n = 3 in sham, n = 5 in other groups). Ten high magnification fields (×200) of the renal cortex and corticomedullary junction were randomly selected from each mouse. (c) Immunoblotting analysis of cleaved caspase-3 in kidney tissue from CIN model mice sacrificed at 24 h after tail vein injection. GAPDH was used as a loading control (n = 5 in each group). Full length blots are presented in Supplementary Figure 1g. (d) Representative image of TdT-mediated dUTP nick end labeling (TUNEL) staining in the kidney from each group of mice. Scale bar: 100 μm. Graph shows the average number of TUNEL-positive cells (n = 3 in sham, n = 5 in other groups). Ten high magnification fields (×200) of the renal cortex and corticomedullary junction were randomly selected, and the average number of TUNEL-positive cells per field was calculated. Data are means ± S.D. #P < 0.01 (Student's t-test).
