# Supplementary figures and images for: Adipose-derived mesenchymal stem cells cultured in serum-free medium attenuate acute contrast-induced nephropathy by exerting anti-apoptotic effects
Source: Stem Cell Res Ther. 2023 Nov 22;14:337. doi: 10.1186/s13287-023-03553-8 (PMC10664307; doi:10.1186/s13287-023-03553-8)

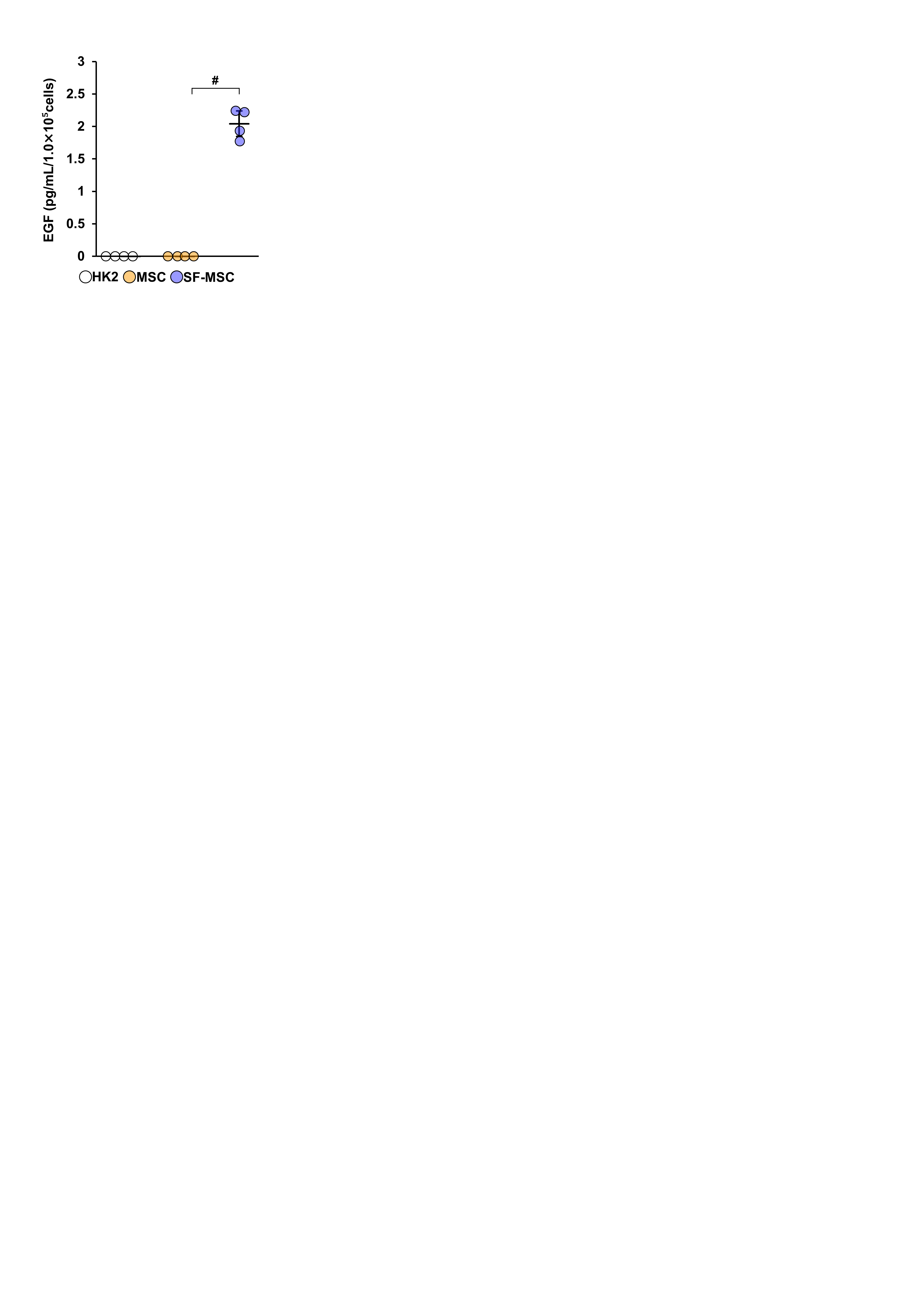

Supplement: Supplementary file 3 — Additional file 3: Fig. S3. Culturing MSCs in serum-free medium increases the secretion of EGF. MSCs, SF-MSCs, and HK-2 cells were cultured in DMEM containing 0.1% FBS for 48 h, and then the culture supernatants were collected as conditioned medium. Epidermal growth factor (EGF) in each conditioned medium were measured by ELISA (n = 4 in each group). Concentrations were normalized to the total number of cells. Data are means ± S.D. P < 0.01 (one-way ANOVA followed by Tukey–Kramer’s post-hoc test). [file 13287_2023_3553_MOESM3_ESM.tif]
